# Editorial: COVID-19 and thrombo-inflammatory responses

**DOI:** 10.3389/fcvm.2023.1231909

**Published:** 2023-06-15

**Authors:** Saravanan Subramaniam, Christoph Reinhardt, Paresh P. Kulkarni, Luca Spiezia

**Affiliations:** ^1^ Pulmonary Center, Department of Medicine, Chobanian & Avedisian School of Medicine, Boston University, Boston, MA, United States; ^2^ Center for Thrombosis and Hemostasis (CTH), University Medical Center of the Johannes Gutenberg-University, Mainz, Germany; ^3^ German Center for Cardiovascular Research (DZHK), Partner Site RhineMain, Mainz, Germany; ^4^ Department of Biochemistry, Institute of Medical Sciences, Banaras Hindu University, Varanasi, India; ^5^ Thrombosis and Hemorrhagic Diseases Unit Medicine, Padova University Hospital, Padova, Italy

**Keywords:** COVID-19, thrombo-inflammation, long covid, VITT, platelets and COVID-19, thrombocytopenia, thromboprophylaxis regimens

**Editorial on the Research Topic** COVID-19 and thrombo-inflammatory responses

## Introduction

The Coronavirus disease 2019 (COVID-19) pandemic caused by severe acute respiratory syndrome Coronavirus-2 (SARS-CoV-2) presents with varying clinical symptoms between individuals. Severe COVID-19 causes pneumonia, acute respiratory distress syndrome (ARDS), cytokine storm, and multi-organ failure (1, 2). Despite the end of the epidemic, COVID-19 is still prevalent, transitioning from a lethal phase to one in which people can become infected without experiencing major symptoms or being hospitalized. COVID-19 is associated with a significant increase in the risk of venous and arterial thromboembolic events in hospitalized patients (3, 4), which is associated with vascular barrier dysfunction, edema, endotheliitis, thrombosis, and inflammatory cell infiltration. Although multiple organ failure in COVID-19 is caused by several mechanisms (5, 6), a hypercoagulation state with the development of micro- and macro- circulatory thrombosis plays a prominent role.

This Research Topic is intended to further understanding of COVID-19 and COVID-19-associated thrombo-inflammatory responses. The current issue has 11 articles, most of which are on thrombocytopenia, thromboprophylaxis regimens, Long COVID, and vaccine-induced immune thrombotic thrombocytopenia (VITT). Many of the articles were written from a clinical standpoint to increase insights into COVID-19 pathophysiology in the setting of prothrombotic response.

## Platelets and COVID-19

Platelets are well-known for their critical contributions to thrombosis and hemostasis (7). During infection, activated platelets adhere to the sub-endothelium, and their hyperactivity results in thrombus formation, leading to arterial ischemia and even pulmonary embolisms. Acetylcholine is known to reduce platelet activation via α7 nicotinic acetylcholine receptors (α7nAChR) (8–10). Accumulating evidence suggests that stimulated platelets generate choline products, which activate the α7nAChR, resulting in a positive anti-inflammatory and anti-thrombotic impact. In this issue, Jankauskaite et al. reviewed platelet functions in COVID-19-associated thrombosis and α7nAChR-mediated anti-inflammatory pathway. Nevertheless, *in vivo* studies are required to validate the significance of α7nAChR in platelet function and whether it might be a possible therapeutic target for reducing platelet hyperreactivity during infection, particularly in COVID-19.

Transforming growth factor-β1 (TGF-β1) functions in maintaining a healthy microvasculature by regulating inflammation, clotting, and wound healing. Platelets are the most abundant source of human TGF-β1 (40–100 times more than other cells), which is stored in its latent form in platelet granules (11). In this issue, Arguinchona et al. have summarized, with caveats, the role of TGF-β1 in thrombosis, inflammation, and immune dysregulation in various diseases, including SARS-CoV-2 infection.

Platelet volume indices (PVI), including mean platelet volume (MPV), platelet distribution width (PDW) and platelet-large cell ratio (P-LCR), are considered useful predictors of thrombotic events (12, 13). In the issue, Daniels et al. systematically evaluated the usefulness of PVI as clinical biomarkers for COVID-19 prognosis and as early predictors for severity and mortality in COVID-19. They found that due to the variability in results, it was difficult to conclude whether COVID-19 patients with elevated PVI are more likely to develop severe illness or are at higher risk of mortality.

Platelets are activated during COVID-19 and participate in thrombo-inflammatory responses (14–16). RNA-Seq has indicated both direct and indirect impacts of SARS-CoV-2 infection (e.g., mediators, aberrant antibodies) on the platelet transcriptome of critically ill COVID-19 patients (16). Due to conflicting reports (17, 18), the exact molecular mechanisms behind the direct activation of platelets during SARS-CoV-2 infection remain largely unknown and are likely multifactorial. Cappelletto et al. screened >3,000 FDA/EMA approved drugs and identified Niclosamide and Clofazimine as the most effective at suppressing Spike-induced TMEM16 activation. Spike induced a marked procoagulant phenotype in platelets, by enhancing Ca^2+^ flux, phosphatidylserine externalization on the platelet outer cell membrane, and thrombin generation which were inhibited by Niclosamide and Clofazimine.

## Fibrin and COVID-19

Coagulation results in an insoluble clot of crosslinked fibrin. Fibrin monomers (FM) have been proposed as a diagnostic marker of DIC (19) and a predictor of thrombosis and/or a hypercoagulable state earlier than D-dimer (20). In healthy individuals, FM levels are very low in peripheral blood, often below the detection limit. In this issue, Smadja et al. evaluated the relationship between FM and COVID-19 mortality in hospitalized patients. FM levels >7 μg/ml were used as lower cut-off and were monitored during initial hospitalization to predict COVID-19 outcomes. During the first 9 days of hospitalization 37% of patients had positive FM at least once; these patients had higher in-hospital mortality (*p* = 0.001), indicating that monitoring fibrin monomers might be a prognostic tool in moderate-to-critically ill COVID-19 patients.

## Thrombocytopenia and prophylactic regimens in COVID-19

High-quality evidence from meta-analyses and randomized controlled trials exploring the clinical outcomes of several preventive regimens in critically ill patients has resulted in contradictory findings (21–23). The anticipated benefit of increasing the anticoagulant dosage is still debated. Rychlíčková et al. in a case study, reported that Fondaparinux can be considered a reasonable and affordable anticoagulant, without a high risk of bleeding, in patients on extracorporeal membrane oxygenation (ECMO). Similarly, Alrashed et al. performed a retrospective cohort analysis (811 patient records) on standard, intermediate, and high anticoagulation dosage regimens in critically ill COVID-19 patients. There were no statistically significant differences in overall in-hospital mortality between the standard-dose and the intermediate-dose groups [51 vs. 53.4%; aHR = 1.4 (95% CI: 0.88–2.33)] or standard-dose and high-dose groups [51 vs. 61.1%; aHR = 1.3 (95% CI: 0.83–2.20)]. The intermediate- and high-dose groups experienced the same frequency of major bleeding episodes as the standard dose group. Thus, these findings recommend standard-dose as the preferred regimen for COVID-19-patients.

## Long COVID-19

The older the patients, the more likely they are to succumb to COVID-19 due to immunological dysfunction (24) and comorbidities (25), such as obesity. Many cohort and case-control studies have shown high body mass index (BMI) as a risk factor for disease severity and mortality in COVID-19 patients (26). In this issue, Xiang et al. review suggest that the intersection of obesity and Long COVID, and persistent viral presence, long-term inflammation, micro clots, and hypoxia may contribute to the development of persistent symptoms, and that patients with obesity are uniquely susceptible to Long COVID.

Jaeger et al. in their perspective, propose that acute and Long COVID patients may benefit from treatment with Heparin-induced extracorporeal LDL/fibrinogen precipitation (H.E.L.P.) apheresis, which has been in clinical use for 37 years. H.E.L.P removes microthrombi without causing bleeding, enhances oxygen supply to the capillaries, lowers cytokine storm, and removes precursors of the procoagulant and fibrinolytic cascade.

## Vaccines and thrombosis in COVID-19

Vaccination has been the most promising strategy for combating the COVID-19 pandemic. Antibodies that recognize platelet factor 4 (PF4, also known as CXCL4) bound to platelets caused VITT (27). Cari et al. conducted a meta-analysis of several adenovirus-based vaccinations and their incidence of VITT, non-VITT thrombosis, and arterial events. Although rare, recipients of the Vaxzevria and Jcovden vaccinations had a greater incidence of VITT compared to Comirnaty, implying a link between these occurrences and the adenovirus-based vaccines. The authors hypothesize that the venous and arterial thromboses observed with adenovirus-based vaccines and in absence of thrombocytopenia are due to the combination of at least three triggering factors, all of which may be involved in vascular inflammation and coagulation and suggest that it is independent of anti-PF4 antibodies. Likewise, Jevtic et al. present an update on the clinical diagnosis of VITT and a comprehensive assessment of VITT epidemiology, and similarities and differences between HIT and VITT. According to the review, HIT and VITT antibodies bind to distinct locations on PF4. Furthermore, diagnostic tests established for HIT frequently produce false-negative findings for VITT and should not be employed as a VITT diagnostic test.

## Conclusions

In conclusion, this special issue (perspective, case report, research findings, selective reviews, and meta-analysis) highlights the importance of COVID-19 and COVID-19-associated thrombocytopenia, thromboprophylaxis regimens, Long COVID, and VITT. Over the past three years, our understanding of COVID-19-associated prothrombotic mechanism is slowly resolving. COVID-19 severity is heavily influenced by co-morbidities. Accumulating evidence reveals that even after recovery, those who had COVID-19 experience ongoing cardiovascular issues such coagulopathy or bleeding disorders. Researchers are also learning more about how new variants could potentially affect Long COVID. We are still investigating to what extent certain groups are at higher risk, and if different groups of people tend to experience different types of Long COVID complications. Further close monitoring of post-COVID conditions will aid in our understanding of Long COVID and how healthcare providers might treat or support people suffering from these long-term impacts.
